# Integrative analysis of multi-omics data for identifying multi-markers for diagnosing pancreatic cancer

**DOI:** 10.1186/1471-2164-16-S9-S4

**Published:** 2015-08-17

**Authors:** Min-Seok Kwon, Yongkang Kim, Seungyeoun Lee, Junghyun Namkung, Taegyun Yun, Sung Gon Yi, Sangjo Han, Meejoo Kang, Sun Whe Kim, Jin-Young Jang, Taesung Park

**Affiliations:** 1 Interdisciplinary program in Bioinformatics, Seoul National University, Seoul, Korea; 2 Department of Statistics, Seoul National University, Seoul, Korea; 3 Department of Mathematics and Statistics, Sejong University, Seoul, Korea; 4 Immunodiagnostics R&D Team, IVD Business Unit, New Business Division, SK telecom Co., Seongnam, Korea; 5 Department of Surgery, Seoul National University Hospital, Seoul, Korea

## Abstract

**Background:**

microRNA (miRNA) expression plays an influential role in cancer classification and malignancy, and miRNAs are feasible as alternative diagnostic markers for pancreatic cancer, a highly aggressive neoplasm with silent early symptoms, high metastatic potential, and resistance to conventional therapies.

**Methods:**

In this study, we evaluated the benefits of multi-omics data analysis by integrating miRNA and mRNA expression data in pancreatic cancer. Using support vector machine (SVM) modelling and leave-one-out cross validation (LOOCV), we evaluated the diagnostic performance of single- or multi-markers based on miRNA and mRNA expression profiles from 104 PDAC tissues and 17 benign pancreatic tissues. For selecting even more reliable and robust markers, we performed validation by independent datasets from the Gene Expression Omnibus (GEO) and the Cancer Genome Atlas (TCGA) data depositories. For validation, miRNA activity was estimated by miRNA-target gene interaction and mRNA expression datasets in pancreatic cancer.

**Results:**

Using a comprehensive identification approach, we successfully identified 705 multi-markers having powerful diagnostic performance for PDAC. In addition, these marker candidates annotated with cancer pathways using gene ontology analysis.

**Conclusions:**

Our prediction models have strong potential for the diagnosis of pancreatic cancer.

## Background

The development of early diagnostic biomarkers and innovative therapeutic strategies to prevent the progression of cancers is urgent. However, common biomarker development strategies, based on gene expression alone, have only limited potential to identify novel biomarkers. Due several distinguishing characteristics, microRNAs (miRNAs) have become new potential biomarkers in cancer genetics. miRNAs are small noncoding RNA molecules which "micro-manage" messenger RNA (mRNA) expression by reducing its translation and stability [1]. Recent studies show that in particular, miRNAs play a crucial role in cancer cell proliferation [2], apoptosis [3], angiogenesis [4], metastasis [5], and chemoresistance [6] by changing the expression of both oncogenes and tumor suppressors [7] in pancreatic cancer. These biological roles of miRNAs represent their potential as diagnostic biomarkers for pancreatic cancer.

An important step of estimating the gene-regulatory activity of miRNAs is accurately predicting their targets and monitoring their expression levels. Several computational target prediction tools have been developed, such as TargetScan version 6.2 [8], PITA version hg18 [9], and miRvestigator [10]. However, these *in silico *target prediction tools suffer from high false positive rates because the tools use only sequence complementarity and assume structural stability (following putative assembly) to predict a specific miRNA's target [11]. As miRNA regulatory activation often depends on the distinct tissue being studied (e.g., cancer tissue), the use of condition (i.e., stress, S-phase, etc.)-specific miRNA and mRNA expression data is required to find true miRNA activity [12]. Therefore, the use of miRNAs as potential biomarkers in dismal cancers such as pancreatic cancer remains difficult.

Pancreatic cancer is one of the most hard-to-diagnose and aggressive malignancies, despite increasing knowledge of its etiology [13]. Because of its highly lethal nature and silent symptoms, pancreatic cancer has remained one of the leading causes of cancer-related death [14]. Among the several types of pancreatic cancers, pancreatic ductal adenocarcinoma (PDAC) is the most abundant cancer type which accounts for about 85% of exocrine pancreatic cancers. Although recent advances in gene expression profiling technology, such as microarray and massively parallel sequencing, enable researchers to discover gene-based biomarkers for PDAC diagnosis, there are no highly effective diagnostic markers for PDAC. In order to improve the survival rate of PDAC patients, it is important to identify efficient diagnostic, prognostic, and therapy response markers.

In this study, we performed a novel approach to identify diagnostic markers for PDAC by integrating miRNA and mRNA expression profiles. Using paired miRNA and mRNA expression profiling, we successfully identified promising mRNA and miRNA markers. By determining differential miRNA expression profiles and interaction with their target genes in PDAC, as compared to normal pancreatic tissues, we estimated miRNA expression levels in independent datasets lacking miRNA expression (i.e., having mRNA data only), and validated the diagnostic performance of miRNA marker candidates.

## Results and discussion

**Figure 1:**
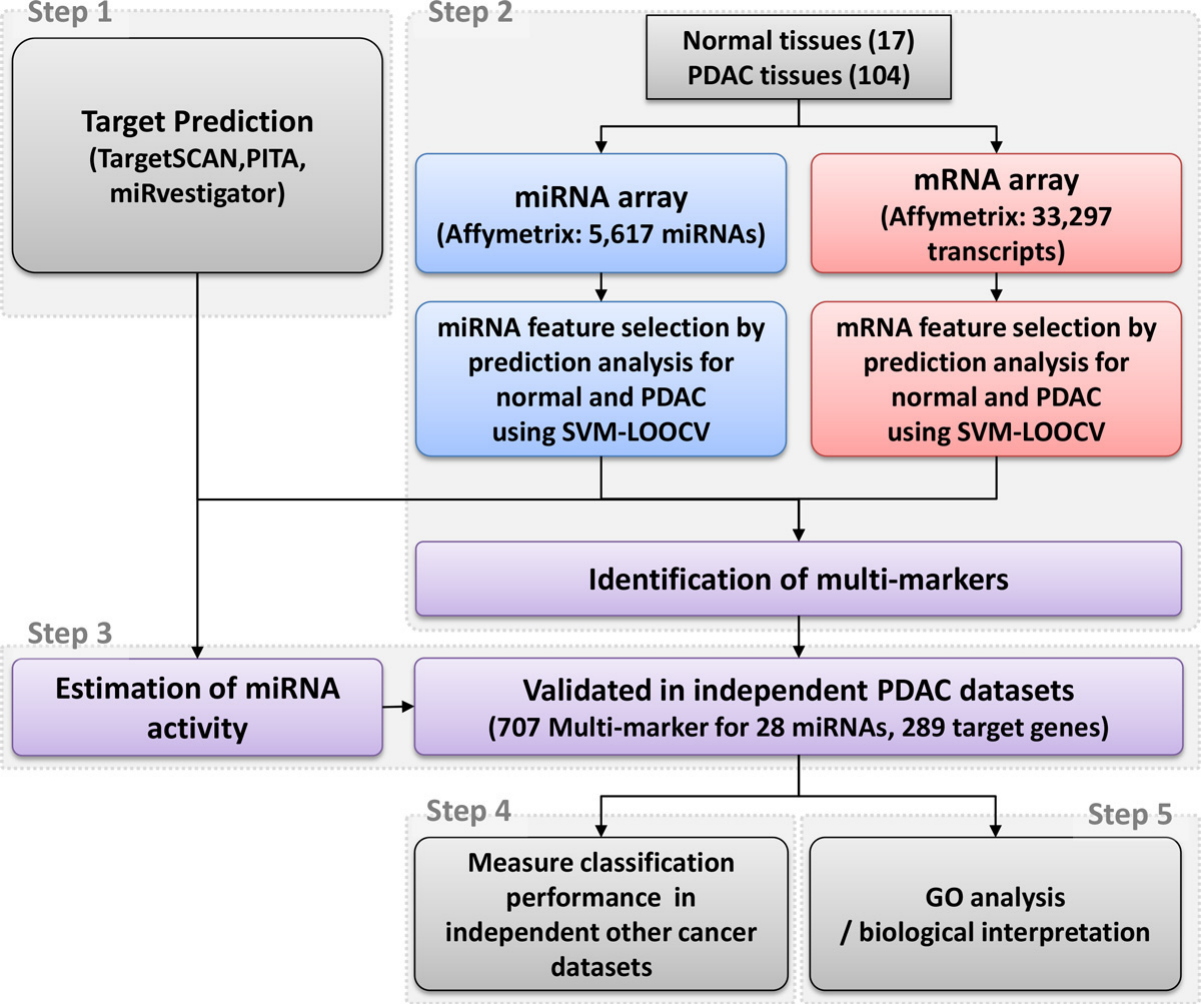
**An analysis scheme of our integrated analysis for PDAC**. 104 PDAC tumor and 17 normal pancreatic tissues were separately analysed for gene and miRNA expression using microarrays. Specific features of miRNAs and mRNAs were modelled by SVM and leave-one-out cross-validation (LOOCV). These were then verified by miRNA target prediction algorithms and finally, validated in independent datasets.

**Figure 2:**
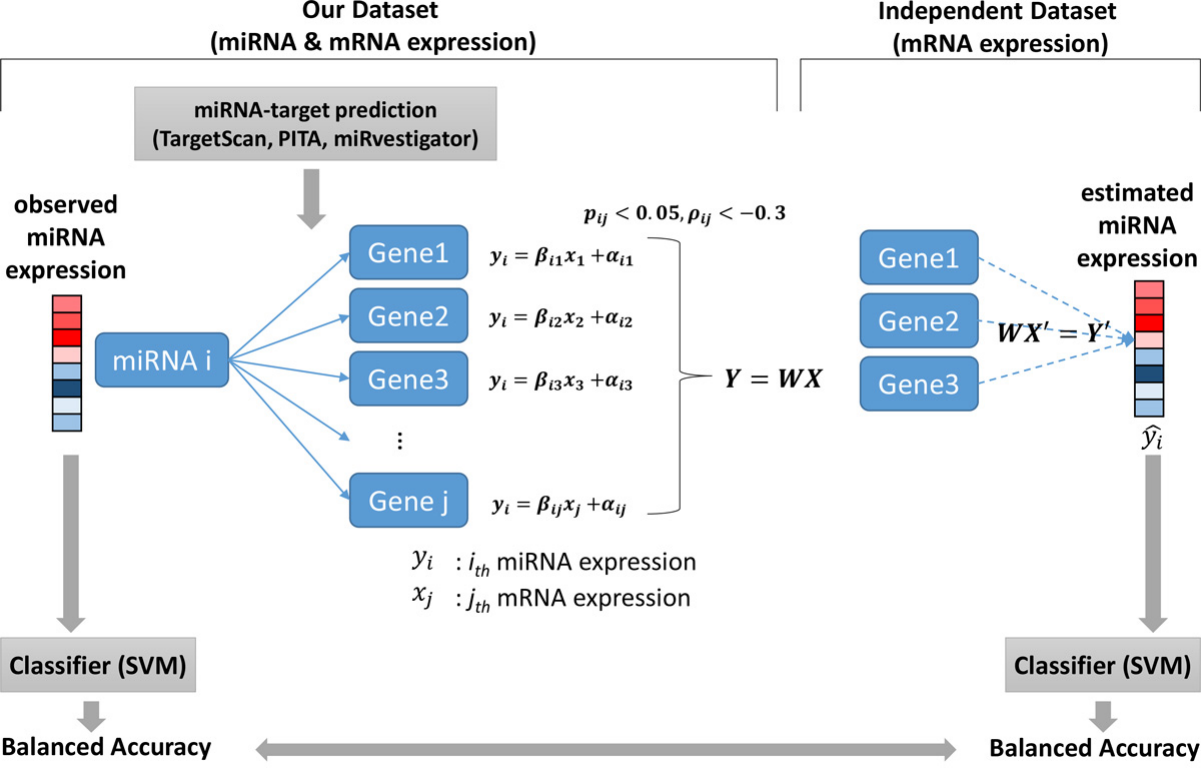
**Estimation scheme miRNA expression**. Based on the predicted targeting activity of specific miRNAs and their targets identified by three miRNA target prediction algorithms, we used linear regression to determine mRNA levels and balanced accuracies for both miRNAs and their specific target transcript mRNAs.

In this section, we firstly identified multi-markers using mRNA and miRNA expression data from 104 PDAC tissues and 17 benign pancreatic tissues, using support vector machine (SVM) classification and leave-one-out cross-validation (LOOCV). Then, using miRNA target interactions constructed using publically available target prediction tools, we validated marker candidates in independent datasets to select more reliable markers. In the case of independent datasets lacking miRNA expression, we used estimated miRNA activity for validation (based on the expression levels of the miRNA target mRNA transcripts). After validation of the selected candidates, we used other cancer datasets to evaluate and annotate their functions, as shown in Figures 1 and 2.

### Identification of multi-marker candidates from PDAC expression data

For identification of multi-marker candidates for PDAC, we used miRNA and mRNA expression data from 121 total pancreatic tissues of 104 PDAC tumors and 17 benign tissues [15]. To prevent overfitting of imbalanced data, LOOCV and SVM with sample class weights were applied, as described in the Methods section. After evaluation analysis using PDAC and independent datasets, we identified 705 multi-markers for 27 miRNAs, and 289 genes for PDAC diagnosis.

Table 1 shows the 39 identified multi-markers with high accuracy (BAs > 0.85 and AUC > 0.85 in our dataset) for diagnosis of PDAC in our training datasets and independent datasets. Specifically, miR-107 was upregulated in PDAC, and miR-107 was recently found to be silenced by promoter DNA methylation in pancreatic cancer [16]. However, DNA demethylation events could induce miR-107 expression showing that epigenetic mechanisms regulating miRNA levels may be involved in pancreatic carcinogenesis. Likewise, miR-135b was reported as a biomarker for PDAC [17], ovarian cancer, and colon cancer [18], in which it promotes proliferation, invasion, and metastasis [19], and miR-135b was similarly upregulated in our findings. By contrast, downregulation of miR-148a was reported in pancreatic, bladder, and lung cancers, and miR-148a was preventative of tumor angiogenesis and cancer progression [20]. miR-21 is also a well-known potential biomarker for diagnosis, prognosis, and chemosensitivity of pancreatic cancer. As most miR-21 targets are tumor suppressors, miR-21 is associated with various cancers such as those of the breast, ovary, cervix, colon, lung, liver, brain, esophagus, prostate, pancreas, and thyroid [21]. miR-222 has also been reported as differentially expressed in most pancreatic cancers, in which it promotes poor survival rates [22].

**Table 1:** Performance of multi-markers.

miRNA							Target gene							
	**PDAC dataset**			**Independent dataset**				**PDAC dataset**				**Independent dataset**		

**miRNA**	**regulation**	**BA**	**AUC**	**PDAC1**	**PDAC2**	**PDAC3**	**target gene**	**corr^a^**	**p-value^b^**	**BA**	**AUC**	**PDAC1**	**PDAC2**	**PDAC3**

**miR-107**	up	0.859	0.851	0.800	0.729	0.670	**DTNA**	-0.625	1.34E-14	0.936	0.937	0.937	0.795	0.810
							**IFRD1**	-0.593	6.44E-13	0.932	0.988	0.949	0.782	0.550
							**KIAA1324**	-0.636	3.30E-15	0.932	0.975	0.920	0.795	0.762
							**BTG2**	-0.629	8.12E-15	0.917	0.982	0.800	0.705	0.550
							**NTRK2**	-0.499	4.83E-09	0.889	0.905	0.823	0.705	0.772
							**VTCN1**	-0.309	5.39E-04	0.880	0.748	0.829	0.705	0.720
							**SGK1**	-0.451	1.85E-07	0.871	0.852	0.817	0.667	0.550
							**ATP8A1**	-0.427	9.36E-07	0.864	0.882	1.000	0.769	0.678
							**USP2**	-0.464	7.14E-08	0.864	0.894	0.960	0.744	0.633
							**PHF17**	-0.600	2.80E-13	0.863	0.941	0.954	0.705	0.932

**miR-135b**	up	0.870	0.935	0.869	0.708	0.713	**BACE1**	-0.599	3.18E-13	0.941	0.967	1.000	0.821	0.786
							**DTNA**	-0.525	5.24E-10	0.936	0.937	1.000	0.795	0.810
							**PELI2**	-0.528	4.08E-10	0.927	0.973	1.000	0.769	0.772
							**VLDLR**	-0.635	4.25E-15	0.922	0.969	1.000	0.756	0.741
							**RRBP1**	-0.388	1.03E-05	0.913	0.995	1.000	0.821	0.550
							**MKNK1**	-0.603	1.88E-13	0.902	0.953	1.000	0.744	0.786
							**BCAT1**	-0.524	6.04E-10	0.893	0.939	1.000	0.859	0.713
							**SEMA6D**	-0.498	5.38E-09	0.893	0.904	1.000	0.769	0.762
							**ATP8A1**	-0.437	4.95E-07	0.864	0.882	1.000	0.769	0.678
							**PHF17**	-0.575	4.54E-12	0.863	0.941	1.000	0.705	0.932

**miR-148a**	down	0.927	0.956	0.897	0.788	0.688	**SLC2A1**	-0.486	1.41E-08	0.962	0.987	0.914	0.756	0.550
							**MBOAT2**	-0.404	3.96E-06	0.929	0.951	0.926	0.872	0.869
							**TRAK1**	-0.371	2.60E-05	0.905	0.973	0.863	0.692	0.793
							**SULF1**	-0.494	7.54E-09	0.878	0.864	0.800	0.923	0.755
							**KLF5**	-0.425	1.10E-06	0.870	0.870	0.926	0.769	0.835
							**LRCH1**	-0.312	4.63E-04	0.865	0.916	0.909	0.654	0.772
							**ETV1**	-0.325	2.57E-04	0.855	0.875	1.000	0.846	0.724

**miR-21**	up	0.897	0.925	0.903	0.725	0.687	**DTNA**	-0.559	2.28E-11	0.936	0.937	0.937	0.795	0.810
							**IFRD1**	-0.532	2.80E-10	0.932	0.988	0.949	0.782	0.550
							**BTG2**	-0.648	6.89E-16	0.917	0.982	0.800	0.705	0.550
							**BCAT1**	-0.551	5.04E-11	0.893	0.939	0.903	0.859	0.713
							**NTRK2**	-0.444	2.92E-07	0.889	0.905	0.823	0.692	0.772
							**LIFR**	-0.596	4.64E-13	0.888	0.964	0.903	0.769	0.918
							**ACAT1**	-0.511	1.81E-09	0.875	0.830	1.000	0.795	0.550
							**PHF17**	-0.609	1.03E-13	0.863	0.941	0.954	0.705	0.932
							**SNTB1**	-0.449	2.21E-07	0.855	0.802	1.000	0.769	0.585

**miR-222**	up	0.924	1.012	0.869	0.736	0.759	**CXCL12**	-0.452	1.69E-07	0.932	0.970	0.851	0.705	0.932

**miR-34a**	up	0.908	0.912	0.806	0.742	0.670	**DTNA**	-0.447	2.43E-07	0.936	0.937	0.937	0.795	0.810
							**BCAT1**	-0.514	1.46E-09	0.893	0.939	0.903	0.859	0.713

^a.^correlation coefficient between miRNA mRNA expression. ^b.^p-value from linear regression with miRNA and mRNA expression.

In Table 2, 27 miRNAs were identified for efficacy in the diagnosis of PDAC. Of these, 22 were previously known to be differentially expressed in pancreatic cancer [7]. However, miR-941, miR-28, mir-487a, mir-299, and mir-503 have never been reported in pancreatic cancer.

**Table 2:** Performances of selected 27 miRNAs.

	PDAC dataset				Independent PDAC dataset		
**miRNA**	**regulation**	**# target genes**	**BA**	**AUC**	**PDAC1**	**PDAC2**	**PDAC3**

miR-148a	down	18	0.927	0.956	0.897	0.788	0.688
miR-222	up	4	0.924	0.962	0.869	0.736	0.759
miR-100	up	11	0.923	0.957	0.794	0.734	0.656
miR-216b	down	4	0.922	0.972	0.777	0.748	0.702
miR-155	up	24	0.912	0.949	0.726	0.740	0.635
miR-203	up	74	0.899	0.921	0.703	0.717	0.676
miR-23a	up	136	0.898	0.987	0.703	0.726	0.685
miR-21	up	33	0.897	0.925	0.903	0.725	0.687
miR-130b	down	20	0.897	0.981	0.771	0.762	0.654
miR-196b	up	1	0.890	0.868	0.789	0.738	0.669
let-7i	up	29	0.883	0.948	0.720	0.746	0.681
miR-1825	down	8	0.881	0.833	0.760	0.745	0.633
miR-135b	up	13	0.870	0.935	0.869	0.708	0.713
miR-941	up	1	0.864	0.849	0.749	0.760	0.553
miR-28	up	20	0.860	0.898	0.749	0.744	0.685
miR-107	up	40	0.859	0.851	0.800	0.729	0.670
miR-145	up	25	0.859	0.892	0.743	0.717	0.666
miR-34a	up	2	0.855	0.811	0.777	0.753	0.679
miR-31	up	5	0.851	0.840	0.811	0.739	0.722
miR-103a	up	39	0.843	0.815	0.737	0.731	0.670
miR-487a	up	3	0.839	0.830	0.720	0.759	0.685
miR-299	up	5	0.836	0.782	0.743	0.724	0.658
miR-503	up	6	0.824	0.830	0.800	0.714	0.683
miR-133b	up	2	0.817	0.831	1.000	0.705	0.657
miR-150	up	1	0.811	0.896	0.806	0.673	0.720
miR-212	up	52	0.810	0.736	0.714	0.732	0.670
miR-92a	up	8	0.806	0.774	0.880	0.727	0.634

**Figure 3:**
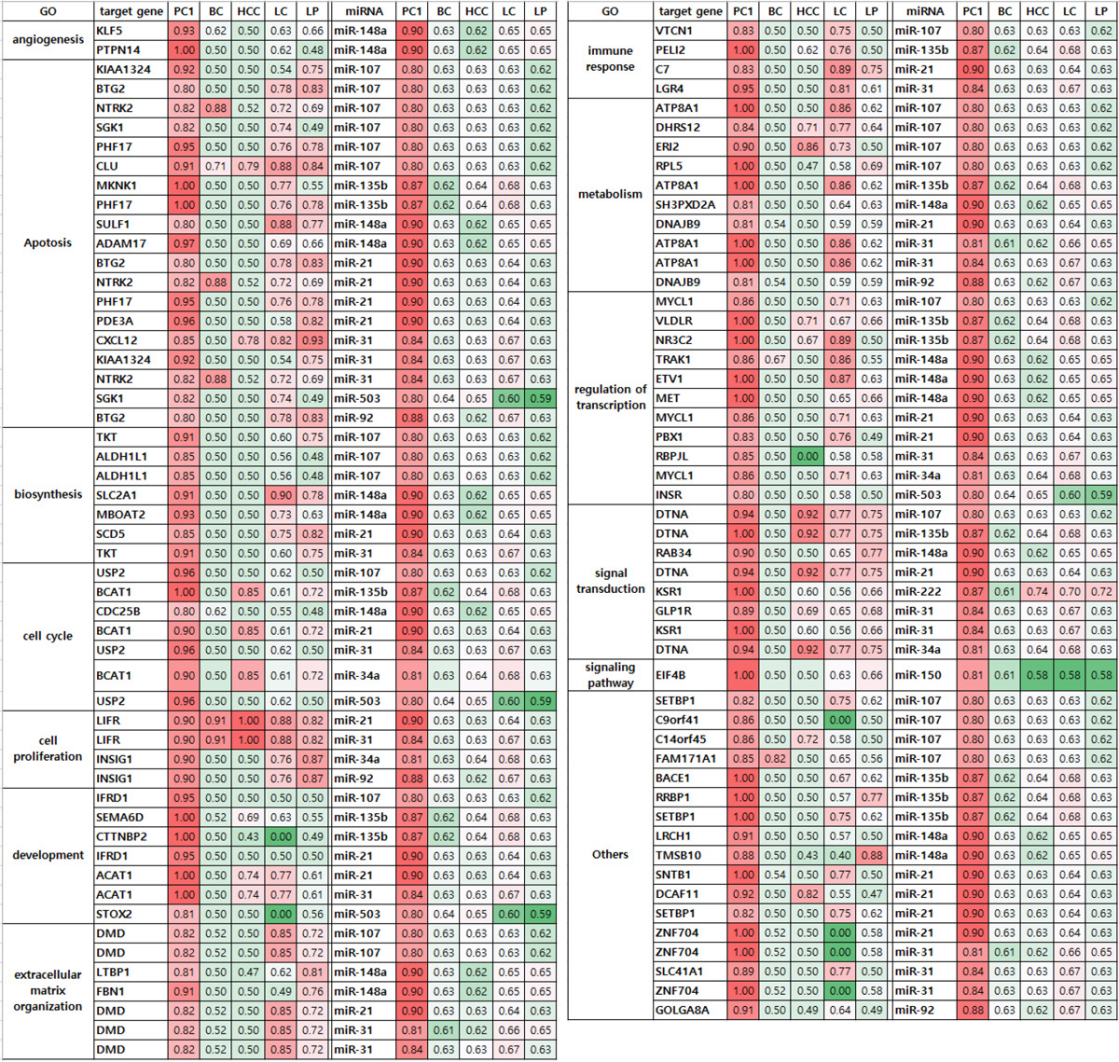
**miRNA-target gene network and Gene ontology**. Blue diamond is miRNA. Circle node is gene. Red circle node is gene with gene ontology related with cancerization such as apoptosis, angiogenesis, cell proliferation, blood vessel development, transcriptional regulation, and immune response.

Out of 289 target genes, 142 were coregulated by more than one miRNA. Table 3 lists 17 target genes that were coregulated by more than 6 miRNAs. Although there are complex interactions between these target genes and miRNAs, their expression direction was required to be negatively correlated (e.g., miRNAs upregulated and targets downregulated) for PDAC vs. normal conditions in miRNA-target gene network (Figure 3). The function of most co-regulated target genes correlated with cancer metabolism and cancer progression, through such processes as attenuated apoptosis, abnormal development, angiogenesis, and transcriptional dysregulation.

**Table 3:** Coregulated target genes.

Target gene	GO	No. of miRNAs	miRNAs
DTNA	signal transduction	12	let-7i, miR-103a, miR-107, miR-135b, miR-203, miR-212, miR-21, miR-222, miR-223, miR-23a, miR-299, miR-34
NTRK2	Apoptosis	11	let-7i, miR-103a, miR-107, miR-203, miR-212, miR-21, miR-222, miR-223, miR-23a, miR-299, miR-31
PHF17	Apoptosis	11	let-7i, miR-103a, miR-107, miR-135b, miR-145, miR-155, miR-21, miR-212, miR-21, miR-222, miR-23a
DMD	extracellular matrix organization	9	let-7i, miR-103a, miR-107, miR-155, miR-203, miR-212, miR-21, miR-223, miR-31
SEMA6D	development	9	miR-103a, miR-107, miR-135b, miR-212, miR-222, miR-23a, miR-31, miR-503, miR-92a
EPB41L4B	actomyosin structure organization	9	let-7i, miR-103a, miR-107, miR-203, miR-212, miR-23a, miR-31, miR-487a, miR-503
BCAT1	cell cycle	9	let-7i, miR-135b, miR-145, miR-155, miR-196b, miR-203, miR-21, miR-28, miR-34
FAM13A	signal transduction	8	miR-203, miR-212, miR-21, miR-222, miR-223, miR-23a, miR-34, miR-487a
GOLGA8A		8	miR-100, miR-203, miR-203, miR-223, miR-223, miR-23, miR-23a, miR-92a
ADHFE1	metabolism	7	let-7i, miR-203, miR-222, miR-223, miR-23a, miR-28, miR-31
ARHGAP24	angiogenesis	7	miR-103a, miR-107, miR-145, miR-203, miR-21, miR-223, miR-23a
ATP8A1	metabolism	7	miR-103a, miR-107, miR-135b, miR-203, miR-23a, miR-28, miR-31
SLC39A14	ion transport	7	miR-155, miR-212, miR-222, miR-223, miR-23a, miR-28, miR-31
ERI2	metabolism	7	let-7i, miR-100, miR-103a, miR-107, miR-203, miR-222, miR-23a
LGR4	immune response	7	let-7i, miR-203, miR-212, miR-222, miR-223, miR-23a, miR-31
SETBP1		7	miR-103a, miR-107, miR-135b, miR-203, miR-21, miR-223, miR-28
INSIG1	cell proliferation	7	miR-100, miR-103a, miR-203, miR-212, miR-222, miR-34, miR-92a

### Estimating the relationship between miRNA activity and miRNA targets

In our previous study [15], we used the average balanced accuracy (BA), i.e., the arithmetic mean of sensitivity and specificity of target-genes, as a metric for miRNA activity performance. In this paper, we modified the estimation algorithm to improve accuracy of miRNA activity (Figure 2). The main difference was that reliable miRNA-target gene relationships were determined by testing pancreatic cancer datasets for estimating miRNA activity.

Using GSE32688 dataset [23] with both mRNA expression and miRNA expression, we evaluated our current and previous miRNA estimation algorithm by comparing the estimated and observed BAs of specific miRNAs. The mean-squared errors were 0.01515 and 0.04877 for our new and previous miRNA estimation algorithms, respectively.

### Diagnostic performance of selected markers in other cancers

**Figure 4:**
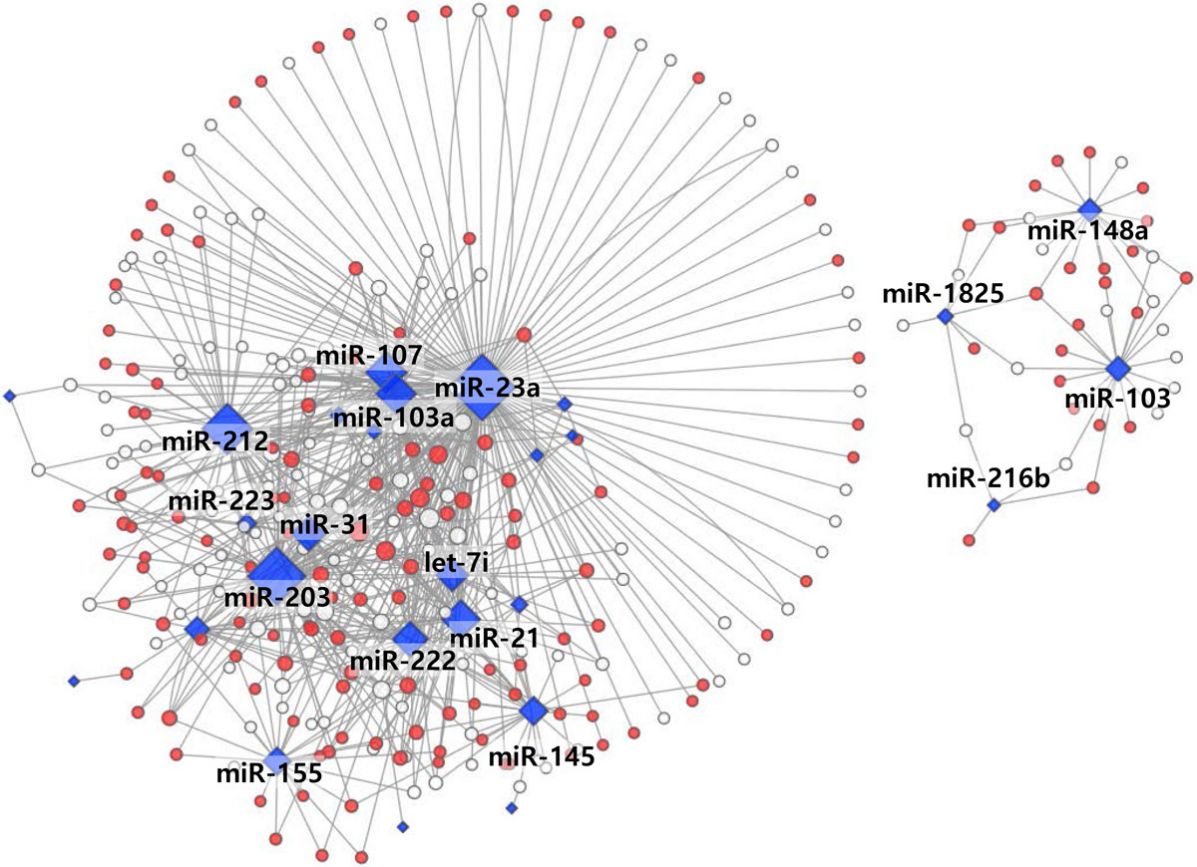
**Diagnostic performance of specific miRNA target genes in other (i.e., non-PDAC) cancers**.

Using our selected PDAC multi-markers, we evaluated their diagnostic performance in lymphoma and breast, hepatocellular, and lung cancers. All independent datasets were collected from the GEO. Figure 4 presents our selected multi-markers for the four other cancers. Most miRNA markers showed weak association with other cancers (besides PDAC).

## Conclusion

In conclusion, we developed a novel single and multi-marker identification approach for PDAC diagnosis by analyzing integrated mRNA and miRNA gene expression profiles. To overcome overfitting of imbalanced data, we applied a SVM model with sample class weights and cross-validation, based on sample partitioning in our dataset and independent datasets. Finally, we identified 705 multi-markers for 27 miRNAs and 289 genes as promising potential biomarkers for pancreatic cancer.

## Methods and materials

### Expression profile datasets

To identify multi-markers in pancreatic cancer, we used mRNA and miRNA expression data from 104 PDAC patients and 17 normal pancreatic patients, following surgery for kidney stones and non-malignant pancreatic disease at Seoul National University Hospital (SNUH) (The detailed experiment and pre-processing steps are described in [15]). All human subjects studies were approved by the Institutional Review Board of Seoul National University Hospital. In this dataset, mRNA and miRNA expression levels were profiled on Affymetrix (Santa Clara, CA, USA) HuGene 1.0 ST (33,297 probes) arrays and Affymetrix GeneChip miRNA 3.0 (25,016 probes) arrays, respectively. We used 5,617 human miRNA probes, out of 25,016 probes, on the Affymetrix GeneChip miRNA 3.0 array.

For validation with independent datasets of selected multi-marker candidates, we collected expression datasets for PDAC (GSE32688 [23], GSE15471 [24], and GSE16515 [25]), lymphoma (LP; GSE14879 [26]), breast cancer (BC; GSE10780 [27]), hepatocellular carcinoma (HCC; GSE6764 [28]), and lung carcinoma (LC; GSE19188 [29]) from the Gene Expression Omnibus (GEO) [30]. All collected expressed data were performed using quantile normalization and RMA normalization by R package.

### miRNA and mRNA biomarker identification for diagnosis of pancreatic cancer

We developed a novel approach to identify candidate mRNA and miRNA multi-markers for PDAC. The schematic workflow of our pipeline is depicted in Figure 1. Paired miRNA and mRNA expression, and miRNA-mRNA networks were integrated to predict performance for diagnosis of PDAC. This approach is composed of five steps. First, the relationships between miRNA and its target genes were constructed by miRNA target prediction tools. Second, mRNA and miRNA biomarker candidates were detected using our PDAC expression data. In the third step, mRNA and miRNA biomarker candidates were validated by independent datasets. Fourth, diagnostic performances of the validated marker candidates were checked in other cancers. Finally, in the last step, the biological functions of the validated marker candidates were annotated.

### Step 1: Prediction of miRNA-target gene interaction

Although many miRNA studies have been performed, only a few miRNA targets have been well validated. To collect reliable miRNA-target relationships covering almost all miRNAs, we employed several *in silico *prediction algorithms. First, we used all validated target information for 567 miRNAs from miRTarBase 4.0 [31], and predicted target information for 2,735 miRNAs from three miRNA target prediction methods such as TargetScan version 6.2 [8], PITA version hg18 [9], and miRvestigator [10]. These three prediction methods were evaluated as reliable methods in [32]. In this paper, we used 1,357,560 miRNA-target relationship data for 2,735 miRNAs and 18,505 targeted genes. For detecting more reliable miRNA-target relationships for specific conditions such as PDAC, only negatively correlated expressed target genes (correlation coefficient < -0.3 and p-value < 0.05 using linear regression) were chosen (Figure 2). Finally, 33,422 miRNA-target relationship data points, for 1,176 miRNAs and 6,424 targeted genes, were used in this study.

### Step 2: Identification of multi-marker candidates with PDAC data

To identify multi-marker candidates, we focused on classification performance with PDAC tissues and benign tissues. In this step, support vector machine (SVM) was applied for qualitative classification evaluated with leave-one-out cross validation (LOOCV). In consideration of our imbalanced sample size (i.e., having many more cancer than benign sample datasets), SVM was employed with sample class weights (*α_cancer _*= 1 and *α_normal _*= 6.117647) [33]. BA, area under the curve (AUC), and p-values from the permutation tests were used for assessing the performance of each prediction model. Using LOOCV, we calculated BA and AUC values from the prediction accuracies of each marker in the testing dataset. BA is defined as an average of sensitivity and specificity, and is a more appropriate evaluation measure for imbalanced datasets than conventional accuracy (i.e., the proportion of the true results among the number of total test datasets). The permutation p-values were calculated from empirical null distribution of BAs by 1 × 10^6 ^sample permutations for markers with high BAs.

Using the miRNA and mRNA target relationships generated in step 1, 1504 multi-markers for 217 genes and 56 miRNAs were selected with BAs > 0.8, AUC > 0.8, and Bonferroni adjusted p-values < 0.05 for genes and miRNAs, respectively.

### Step 3: Evaluation of prediction performance in independent PDAC datasets

To avoid selection of markers with specific data-dependency or specific platform-dependency, all identified single or multi-markers were evaluated using three public, independent PDAC datasets collected from the GEO [30] (Table 2). Of the three, PDAC dataset1 had both mRNA and miRNA expression microarray profiles from GSE32688 [23], while PDAC dataset2 and dataset3 had only mRNA expression profiles using microarray data from GSE15471 [14] and GSE16515 [25]. To select reliable and robust miRNA-target gene multi-markers, miRNAs and their putative target genes having negatively correlated expression, and BAs > 0.7 in PDAC dataset1, were selected.

To validate miRNA prediction performance in the profile datasets (PDAC datasets 2 and 3) containing only mRNA expression, we estimated the expression of specific miRNAs using their predicted miRNA-target gene relationships. In Figure 2, linear regression models were fitted with miRNA and mRNA expression data from the 104 cancer tissues and 17 benign tissues. Then, the expression of the miRNAs of interest was estimated by regression models and its targeted-gene expression data in the independent datasets. Using this estimated miRNA expression, its prediction performance could then be calculated. We extracted the multi-markers with BAs > 0.7 in one or more of the PDAC datasets 2 and/or 3. Finally, after validation with the three independent PDAC datasets, we selected 712 miRNA-target gene multi-markers for 30 miRNAs and 290 genes.

### Step 4: Evaluation of prediction performance in other cancer datasets

To examine the feasibility of repurposing our identified marker candidates for other cancers, we collected other cancer datasets having mRNA expression data for lymphoma [26], breast cancer [27], hepatocellular carcinoma [28], and lung carcinoma [29] from GEO datasets. Based on SVM-LOOCV evaluation analysis, the selected single and multi-markers were evaluated.

### Step 5: Gene ontology analysis and miRNA-mRNA network generation using the identified biomarkers

The targeted genes of the identified multi-markers were annotated for gene ontology pathways/processes (GO) using PANTHER [34]. In this analysis, markers with annotation results with Bonferroni-corrected p-values < 0.05 were selected. Using this GO annotation, miRNA-target gene relationships of identified multi-markers were represented by the network generated by Cytoscape 3.1.1 [35] (Figure 3).

## List of abbreviations used

AUC, Area under curve; BA, Balanced accuracy; BR, Breast cancer; GEO, Gene Expression Omnibus; GO, Gene ontology; HCC, Hepatocellular carcinoma; LC, Lung cancer; LOOCV, Leave-one-out cross-validation; LP, Lymphoma; mRNA, messenger RNA; miRNA, microRNA; PDAC, Pancreatic ductal adenocarcinoma; SVM, Support vector machine; TCGA, the Cancer Genome Atlas;

## Competing interests

The authors declare that they have no competing interests.

## Authors' contributions

MK performed the analysis, and drafted the manuscript. YK performed the analysis of microarray. SL participated in the design of the study. JN, TY, SY and SH performed the microarray experiment. MK, SK and JJ conducted the sample collection and preparation. TP and JJ conceived of the study, and participated in its design and coordination. TP helped to draft the manuscript. All authors write, read and approved the final manuscript.
