# Erratum to: Integrative analysis of multi-omics data for identifying multi-markers for diagnosing pancreatic cancer

**DOI:** 10.1186/s12864-016-3464-x

**Published:** 2017-01-16

**Authors:** Min-Seok Kwon, Yongkang Kim, Seungyeoun Lee, Junghyun Namkung, Taegyun Yun, Sung Gon Yi, Sangjo Han, Meejoo Kang, Sun Whe Kim, Jin-Young Jang, Taesung Park

**Affiliations:** 1 Interdisciplinary program in Bioinformatics, Seoul National University, Seoul, Korea; 2 Department of Statistics, Seoul National University, Seoul, Korea; 3 Department of Mathematics and Statistics, Sejong University, Seoul, Korea; 4 Immunodiagnostics R&D Team, IVD Business Unit, New Business Division, SK telecom Co, Seongnam, Korea; 5 Department of Surgery, Seoul National University Hospital, Seoul, Korea

## Erratum

After publication of this article [1] it was brought to the Editors’ attention that there were inaccurate descriptions of validation data in its abstract and list of abbreviations. For the validation, we used the three datasets from Gene Expression Omnibus (GEO), not the Cancer Genome Atlas (TCGA).

The corrected description for line 8–10 in abstract is as below:

“For selecting even more reliable and robust markers, we performed validation by independent datasets from the Gene Expression Omnibus (GEO) data depository.”

In page 9, the corrected description of list of abbreviations used is as follows:

“AUC, Area under curve; BA, Balanced accuracy; BR, Breast cancer; GEO, Gene Expression Omnibus; GO, Gene ontology; HCC, Hepatocellular carcinoma; LC, Lung cancer; LOOCV, Leave-one-out cross-validation; LP, Lymphoma; mRNA, messenger RNA; miRNA, microRNA; PDAC, Pancreatic ductal adenocarcinoma; SVM, Support vector machine;”
